# Arterio-Venous Thrombosis and Spontaneous Bleeding in COVID-19-Associated Coagulopathy: A Case Report

**DOI:** 10.7759/cureus.27770

**Published:** 2022-08-08

**Authors:** Kafaf S Jalali, Ahmed Basala, Mohamed Habeb

**Affiliations:** 1 Internal Medicine, Prince Mohammed Bin Abdulaziz Hospital / Saudi Commission for Health Specialties, Medina, SAU; 2 Faculty of Medicine, Pulmonology, Ain Shams University, Cairo, EGY; 3 Faculty of Medicine, Pulmonology, Zag University, Zagazig, EGY

**Keywords:** retroperitoneal hematoma, renal artery stenosis, renal artery thrombosis, cerebral venous thrombosis, covid-19

## Abstract

Coronavirus disease 2019 (COVID-19) is caused by severe acute respiratory syndrome which was declared a global pandemic in 2019 causing significant morbidities and mortalities. COVID-19 is a multi-systemic disease and is not primarily limited to the respiratory system. Thrombus formation is one of its distinct features. However, renal complications associated with COVID-19 are rarely reported in the literature due to limited occurrence and research. We report a rare case of right retroperitoneal hematoma in a COVID-19 patient. We report a 51-year-old male patient who was received at the emergency department (ED). The patient was positive for COVID-19 and had a Glasgow coma scale of 12/15. The patient was initially managed on IV anticoagulation due to cavernous sinus thrombosis and was placed on mechanical ventilation which helped him to improve. After two weeks, a sudden drop in hemoglobin was observed. CT scan of abdomen and pelvis showed the presence of a right retroperitoneal hematoma, and right renal artery non-occlusive filling defect. The patient was successfully managed with conservative treatment. Retroperitoneal hematoma although a rare occurrence in COVID-19 patient should be observed and monitored closely in case of bleeding or anemia, as early management and intervention is beneficial.

## Introduction

Coronavirus disease 2019 (COVID-19) is caused by the severe acute respiratory syndrome coronavirus 2 (SARS-CoV-2), a primarily respiratory disease that was declared a pandemic in 2019. It is responsible for approximately 11.5% mortality rate among hospitalized patients [1]. Patients infected with SARS-CoV-2 may be asymptomatic or show minimal symptoms in 80% of cases, while around 10% develop severe respiratory symptoms that progress to acute respiratory distress syndrome [2-3]. Although COVID-19 primarily affects the respiratory system, it also affects other organs such as the gastrointestinal system, the cardiovascular system, the liver, and the kidneys. The severity of the disease is related to how these different organ abnormalities interact with one another. The extensive distribution of angiotensin converting enzyme 2 (ACE2) in the body has been connected to SARS-CoV-2 multiorgan involvement. Moreover, ACE2 expression is most prominent in the ileum and kidneys. Furthermore, new evidence suggests that COVID-19 may have a role in unfavorable renal manifestations such as acute kidney injury, which is linked to the severity of COVID-19 and mortality. The renal manifestations and complications of COVID-19 are not well described due to the availability of sparse data [4].

The true incidence of bleeding in COVID-19 is unknown due to a paucity of data given in the literature, despite the fact that thrombosis is one of the disease's characteristics. In people with COVID-19 infection, thromboembolic events have been observed to occur at a rate as high as 21% with a death rate of 74% [2, 5]. Since patients do not show any clinically obvious symptoms or indicators until significant blood loss has taken place, the diagnosis of retroperitoneal hematomas calls for a high level of clinical suspicion. Stopping or altering anticoagulant treatment and volume resuscitation with fluid and blood products constitute the mainstay care of retroperitoneal hematoma. Small hematomas without displacement of retroperitoneal structures and show also do not require several blood transfusions and can be managed conservatively [6]. We report a rare case of right retroperitoneal hematoma in a COVID-positive patient.

## Case presentation

A 51-year-old male patient presented to the emergency department (ED) by the Red Crescent with a history of high-grade fever, severe headache, left-side weakness, disturbed level of consciousness, and repeated attacks of seizures. The patient is diabetic on oral hypoglycemic agents for the past five years. He is a non-smoker with a body mass index (BMI) of 24.59. At presentation, the Glasgow Coma Scale (GCS) was 12/15 with Eye 3, Motor 5, and Verbal 4. The blood pressure was 110/70 mmHg, pulse 98 beats/min, respiratory rate 35 breaths/min, temperature 38.4°C, and oxygen saturation of 75% on room air. The patient did not respond to oxygen supplementation and started to have respiratory fatigue and was intubated and mechanically ventilated accordingly. Complete blood count, liver and kidney functions along with electrolytes and coagulation were normal except for elevated inflammatory markers (Table 1).

**Table 1:** Laboratory findings of the patient. ESR, erythrocyte sedimentation rate; CRP, C-reactive protein

#	Investigation	Value	Reference value
	ESR	45	1-13 mm/h
	CRP	235	Less than 10 mg/L
	D-Dimer	1000	Less than 0.50
	Ferritin	781	12-300 ng/mL
	Hemoglobin	7	13.8-17.2 g/dL

**Figure 1:**
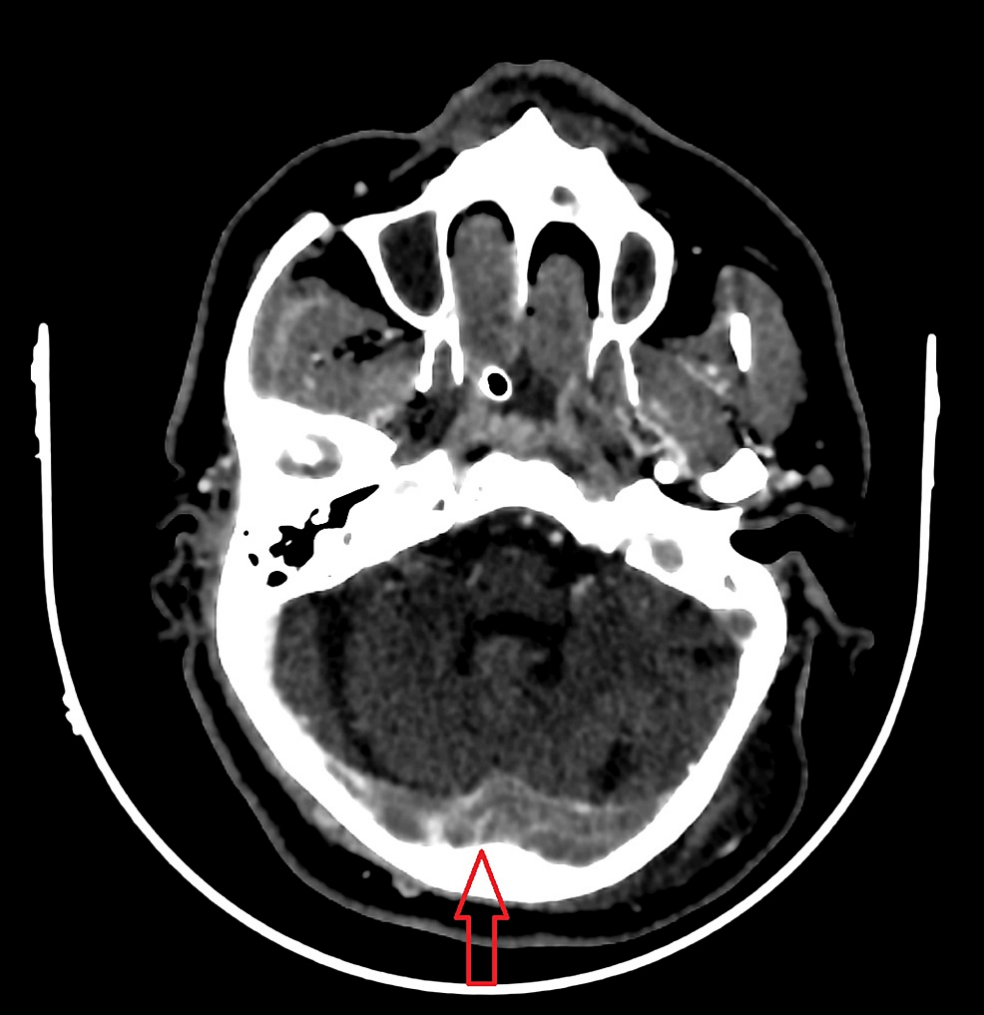
A CT-venogram of the head showing extensive acute dural sinus thrombosis (red arrow).

**Figure 2:**
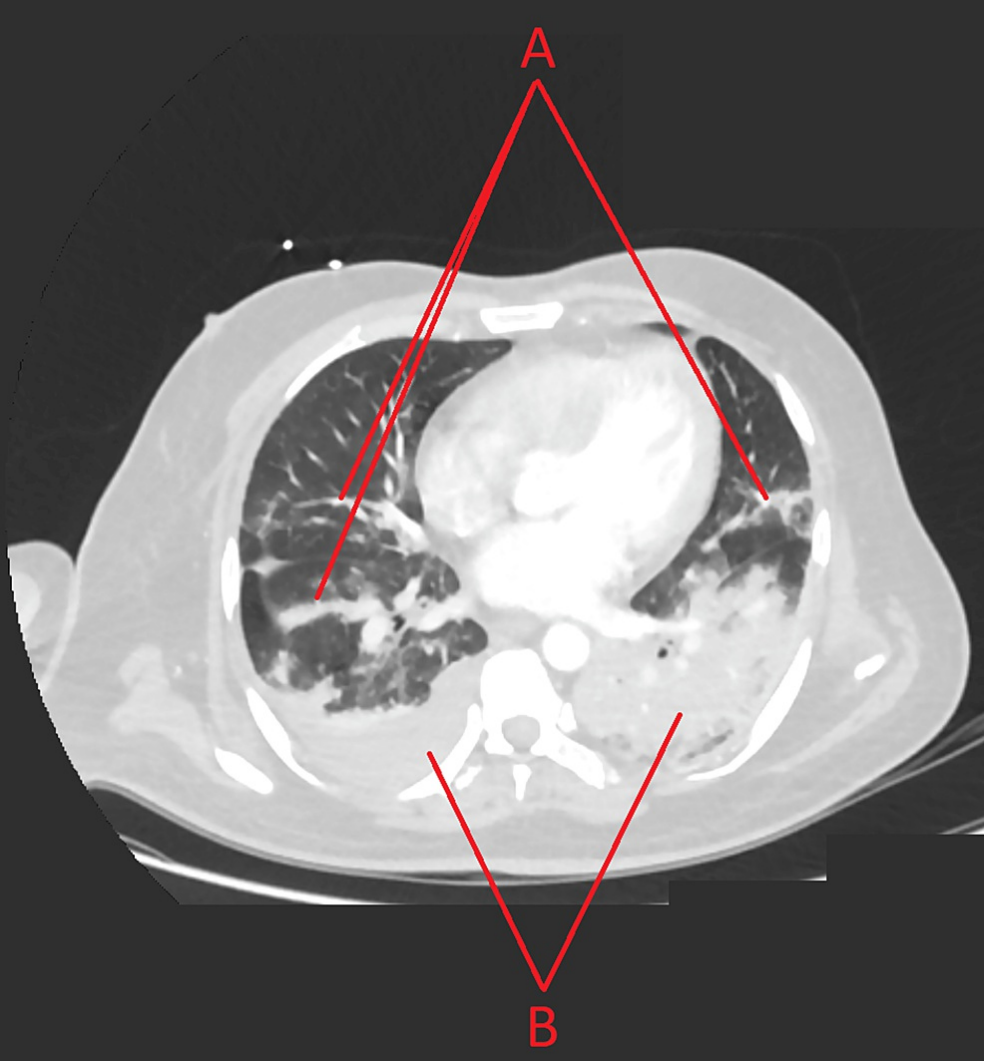
A CT pulmonary angiography. A) Bilateral patchy consolidation denoting an active infection. B) Mild pleural effusion

A CT-venogram of the head revealed extensive acute dural sinus thrombosis, with right frontal and parietal venous infarction (Figure 1). CT-angiogram of the brain was normal with no arterial abnormalities. CT-pulmonary angiography showed bilateral patchy consolidation denoting an active infection with mild pleural effusion with no signs of pulmonary embolism (Figure 2).

**Figure 3:**
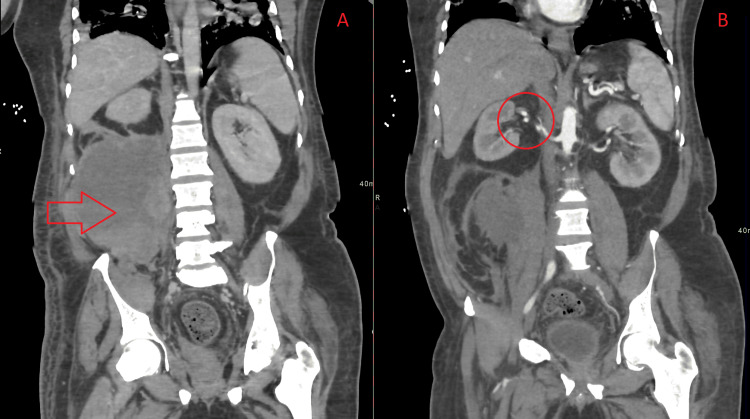
CT of the abdomen and pelvis. A) Right retroperitoneal hematoma (red arrow). B) Right renal artery non-occlusive filling defect (circle).

A nasal swab taken for COVID-19 was positive and the patient was then shifted to the critical care isolation unit. He was started on therapeutic anticoagulation, IV meropenem, methylprednisolone, and hydroxychloroquine. Electrocardiography and echocardiography were performed and showed sinus tachycardia with good left ventricular function, ejection fraction of 60%, no valvular abnormality, no dilatation, and systolic pulmonary arterial pressure was 30 mmHg. In addition, thrombophilia workup was negative for any hematological disorder. After 5 days, the patient was extubated and was saturating well on 10 L simple face mask, with a GCS of 13/15. He continued to improve gradually from the respiratory point until day 14 of admission when a sudden drop of hemoglobin was observed from 13 g/dL to 7 g/dL. Complete blood count, liver and kidney function tests along with coagulopathy tests were performed and were normal. Esophagogastroduodenoscopy was done and showed no active bleeding. CT of the abdomen and pelvis revealed right retroperitoneal hematoma and a right renal artery non-occlusive filling defect (Figure 3). The patient was evaluated by the surgical team who advised conservative management with follow-up and did not recommend surgery. After 20 days, the patient became vitally stable, with an oxygen saturation of 98% on a 5 L face mask and a GCS of 15/15; the patient was retested for COVID-19 and the result was negative after which he was transferred to a medical unit.

He was started on therapeutic subcutaenous enoxaparine 80 mg for 14 days for the cavernous sinus thrombosis. As a result, he developed a sudden drop in hemoglobin but was hemodynamically stable. The patient underwent a CT scan which showed retroperitoneal hematoma. After discussion among the multidisciplinary team regarding the risk of thrombosis and current bleeding, a decision to decrease the dose to 40 mg was made and to treat the hematoma conservatively with close observation of his hemodynamic status as well as his hemoglobin levels. After three weeks, a repeated CT scan showed a decrease in the size of the hematoma. Afterward, the anti-coagulation dose was changed to the original therapeutic one of 80 mg. After 24 days, the patient’s symptoms continued to improve, and his oxygen saturating was 93% on room air with residual left-side weakness. He was discharged on subcutaneous enoxaparin 60 mg every 12 h, levetiracetam 500 mg orally every 12 h, metformin 500 mg orally every 12 h, and advised for regular follow-up at the outpatient department. 

## Discussion

COVID-19 patients commonly develop coagulopathy, which is related to a high rate of thrombotic events and poor outcomes [7]. In patients with COVID-19, biomarkers of coagulation such as D-dimer, fibrinogen, platelet count, interleukin-6 and lymphocyte count, as well as clinical scoring systems, can help predict clinical course, the need for hospital resources such as ICU beds, intubation and ventilator therapy, and patient outcome [8]. Al‐Samkari et al. stated that D-dimer levels are higher in patients with COVID-19 [9]. High incidences of venous thromboembolism and disseminated intravascular coagulation have been reported in early publications [9]. D-dimer levels on admission indicated critical illness, death, bleeding, and thrombotic events. C-reactive protein (CRP) and erythrocyte sedimentation rate (ESR), two inflammatory markers, were linked to thrombosis, and elevated levels of multiple coagulation and inflammatory markers were linked to critical illness and mortality [9]. COVID-19 was linked to the same levels of thrombosis and bleeding in hospitalized individuals with equal levels of serious illness. Bleeding complications, thrombotic complications, critical illness, and death were all predicted by elevated D-dimer values at the time of initial presentation. Meanwhile, thrombosis was linked to inflammatory indicators rather than coagulation measures [10]. Elevated D-dimer levels were observed in our patient also along with raised ESR and CRP levels.

Reported a case of a 51-year-old man who tested positive for COVID-19, his chest X-ray revealed that the patient had bilateral and extensive pulmonary ground-glass opacities and developed diffuse stomach pain. CT of the abdomen and pelvis showed the presence of massive hematoma in the right retroperitoneal space that had displaced the kidney. Active bleeding was detected on the angiography of ileo-lumbar artery. Lee stated that in comparison to non-vascular observations, vascular findings are rather uncommon. Both thrombotic and hemorrhagic adverse events occur in COVID-19 patients, with hemorrhagic events being more common [11]. The most common hemorrhagic complication is hematomas involving the retroperitoneum, abdominal wall, gluteal regions, or upper thigh. Both blockage of major arteries and solid organ infarction, such as the intestinal tract, kidneys, pancreas, and liver, are examples of thrombotic events seen on CT angiography [11]. The CT findings of the abdomen and pelvis of our patient exhibited right retroperitoneal hematoma and right renal artery non-occlusive filling defect that might be due to the anticoagulation therapy.

Arterial thrombosis and venous thromboembolism are two main types of thrombosis that can develop. COVID-19 patients have microthrombi in their alveolar capillaries. Because thrombosis may play a role in the progression of COVID-19, patients with moderate and severe infections should receive prophylactic anticoagulant medication, such as low-molecular-weight heparin or unfractionated heparin. The risk of thrombosis remains significant even with preventative anticoagulant medication. As a result, anticoagulant medication may be needed in conjunction with inflammatory management and vascular endothelial protection [12]. According to growing clinical experience, the management of coagulopathy linked with COVID-19 is extremely difficult and continues to evolve. Due to the close link between inflammation and coagulation, inflammatory parameters such as IL-6, CRP, ferritin, and procalcitonin are particularly useful to stratify patients' thrombotic risk in addition to the recommended laboratory monitoring of coagulopathy like D-dimer, fibrinogen, and platelet count. Given COVID-19's thrombotic burden, low-molecular-weight heparin thromboprophylaxis is now considered a treatment priority, considering the anti-inflammatory effects of this anticoagulant drug, and is suggested by a number of national and international scientific organizations and expert panels. Anticoagulant prophylaxis, on the other hand, should be tailored to each patient's thrombotic risk profile and SARS-CoV-2 disease feature [13]. In our case, the patient was also placed on enoxaparin 60 mg for every 12 h and advised regular follow-up at the outpatient clinic.

## Conclusions

The right retroperitoneal hematoma was observed in our COVID-19 patient. Retroperitoneal hematoma even though is uncommon in COVID-19 patients, should nevertheless not be ignored as a potential source of bleeding, particularly when patients have flank pain, anemia, or hypovolemia symptoms. Close observation and early management may help these patients have a better prognosis.
